# General and abdominal obesity operate differently as influencing factors of fracture risk in old adults

**DOI:** 10.1016/j.isci.2022.104466

**Published:** 2022-05-25

**Authors:** Xiao-Wei Zhu, Ke-Qi Liu, Cheng-Da Yuan, Jiang-Wei Xia, Yu Qian, Lin Xu, Jian-Hua Gao, Xiao-Li Rong, Guo-Bo Chen, David Karasik, Shu-Yang Xie, Hou-Feng Zheng

**Affiliations:** 1 Fudan University, Shanghai 200433, China; 2 Diseases & Population (DaP) Geninfo Lab, School of Life Sciences, Westlake University, Hangzhou, Zhejiang 310024, China; 3 Institute of Basic Medical Sciences, Westlake Institute for Advanced Study, Hangzhou, Zhejiang 310024, China; 4 WBBC Jiangxi Center, Jiangxi Medical College, Shangrao, Jiangxi 334000, China; 5 Department of Dermatology, Hangzhou Hospital of Traditional Chinese Medicine, Hangzhou, Zhejiang 310007, China; 6 WBBC Shandong Center, Binzhou Medical University, Yantai, Shandong 264003, China; 7 Institute for Cell Engineering, The Johns Hopkins University School of Medicine, Baltimore, MD 21205, USA; 8 Clinical Research Institute, Zhejiang Provincial People’s Hospital, Hangzhou Medical College, Hangzhou 310014, China; 9 Azrieli Faculty of Medicine, Bar-Ilan University, Safed 5290002, Israel

**Keywords:** Health sciences, Obesity medicine, Risk factor

## Abstract

To infer the causality between obesity and fracture and the difference between general and abdominal obesity, a prospective study was performed in 456,921 participants, and 10,142 participants developed an incident fracture with follow-up period of 7.96 years. A U-shape relationship was observed between BMI and fracture, with the lowest risk of fracture in overweight participants. The obesity individuals had higher fracture risk when BMD was adjusted, and the protective effect of moderate-high BMI on fracture was mostly mediated by bone mineral density (BMD). However, for abdominal obesity, the higher WCadjBMI (linear) and HCadjBMI (J-shape) were found to be related to higher fracture risk, and less than 30% of the effect was mediated by BMD. By leveraging genetic instrumental variables, it provided additional evidences to support the aforementioned findings. In conclusion, keeping moderate-high BMI might be of benefit to old people in terms of fracture risk, whereas abdominal adiposity might increase risk of fracture.

## Highlights

• Overweight individuals have the lowest risk of fracture compared with other groups
• The protective effect of moderate-high BMI on fracture is mostly mediated by BMD
• The higher WCadjBMI and HCadjBMI are found to be related to higher fracture risk

## Graphical abstract

**Figure:**
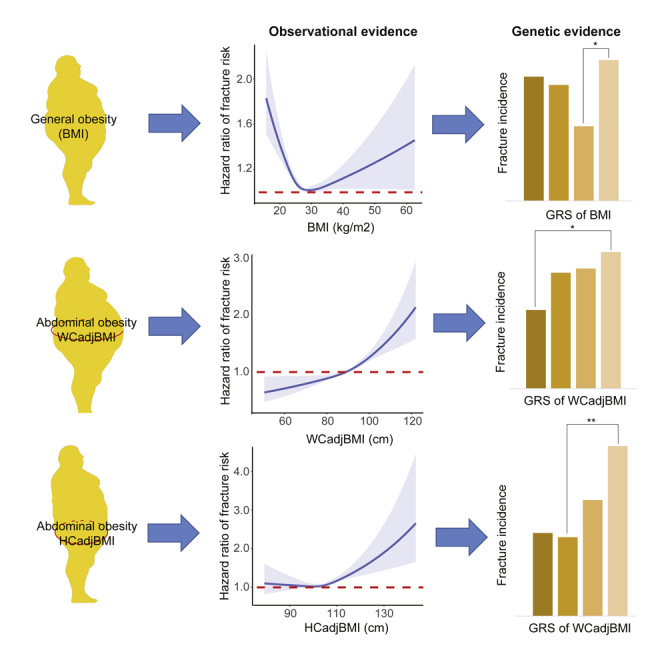

## Introduction

Fractures in older adults are often the precursor of disability, loss of independence, and premature death, seriously affecting their quality of life (Svedbom et al., 2018). As a complex disease, fracture is influenced by both genetic and environmental factors, and dozens of susceptible loci have been identified by genome-wide association studies (GWASs) (Zhu et al., 2021). Many environmental factors were reported to be related to the incidence of fracture, such as smoking (Wu et al., 2016), alcohol intake (Wang et al., 2020), physical activity (Cauley and Giangregorio, 2020), and dietary intakes (Mozaffari et al., 2018). Previous studies have suggested that the increased falling was one of the major risk factors for fracture among older people (Schwartz et al., 2005), and falls account for 87% of all fractures in the elderly (Fife and Barancik, 1985). Besides, low bone mineral density (BMD) was another major risk factor of fracture risk confirmed by mendelian randomization (MR) analyses (Trajanoska et al., 2018).

Obesity was previously deemed to be a protective factor for osteoporosis or brittle fractures because patients affected by obesity have more soft tissue to protect bone tissue (De Laet et al., 2005; Tang et al., 2013). However, recent studies suggested that obesity might increase the risk of certain fracture types (Cao and Picklo, 2015; Scott et al., 2016; Kim et al., 2018). Kim et al. found that overweight might be protective against hip fracture in Asian adults but not obesity, and lower body mass index (BMI) was a risk factor for hip fracture, whereas obesity was associated with an increased risk of hip fracture, particularly in women (Kim et al., 2018). In addition, because abdominal obesity is a surrogate of visceral fat with more endocrinological activities than subcutaneous fat, using different obesity indices would add information to differentiate the role of fat accumulation on bone health (Ibrahim, 2010). In epidemiologic studies, waist circumference (WC) and hip circumference (HC) are used as surrogate indices of abdominal adiposity (Yang et al., 2006; Ahmad et al., 2016). Associations between abdominal obesity indices and fracture were inconsistent; most of the studies found that abdominal obesity increased the risk of fracture (Sogaard et al., 2015; Li et al., 2017; Ofir et al., 2020), whereas few studies reported nonassociation (Benetou et al., 2011) and some studies reported opposite findings between males and females (Laslett et al., 2012; Luo and Lee, 2020).

Although there were some explanations for the controversial findings, leveraging genetic data to infer the causal relationship between exposure and outcome could be additional evidences for association (Xia et al., 2020; Qian et al., 2021). Therefore, in the present study, we firstly conducted a prospective observational study to investigate the relationship between general obesity index (BMI), abdominal obesity indices (waist circumference adjusted for BMI [WCadjBMI], and hip circumference adjusted for BMI [HCadjBMI]) and fracture risk using the UK Biobank dataset. We tried to explore the intermediate role of BMD or falls on the association between obesity indices and fracture. Furthermore, we performed genome-wide association analyses for BMI, WCadjBMI, and HCadjBMI, and tested the causal association between genetically determined obesity indices and fracture.

## Results

### The association of general obesity index with fracture risk

**Figure 1:**
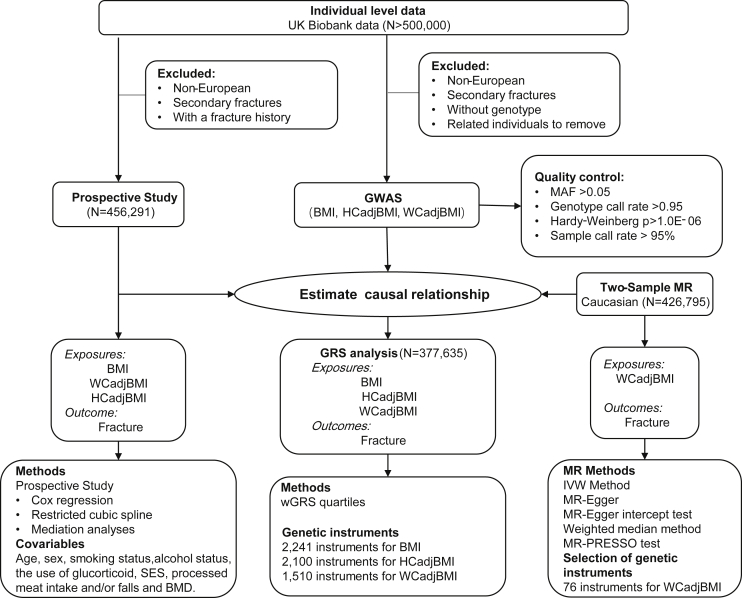
Overview of the study design BMI, body mass index; MR, Mendelian randomization; WCadjBMI, waist circumference adjusted for body mass index; HCadjBMI, Hip circumference adjusted for body mass index; SES, socioeconomic status.

**Figure 2:**
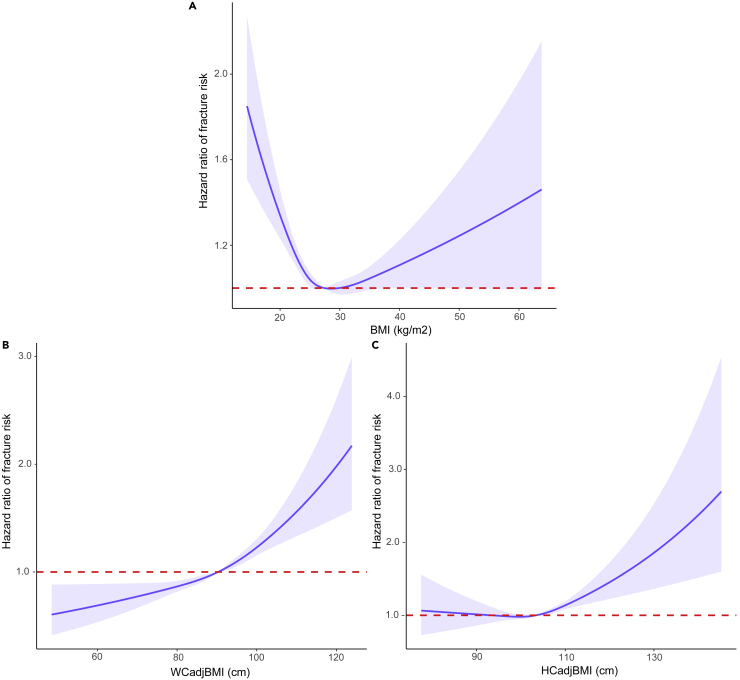
Observational association of obesity-related indices with fracture risk using a restricted cubic spline method based on model 0 bservational association of obesity-related indices with fracture risk using a restricted cubic spline method based on model 0: (A) BMI = 27.4 kg/m^2^; (B) WCadjBMI = 90.29 cm; (C) HCadjBMI = 103.43 cm. In all these analyses, models were adjusted for risk factors for fracture, including age, sex, smoking statue, alcohol drinker status, physical activity and the use of glucocorticoid, socioeconomic status, and processed meat intake. Hazard ratios are indicated by solid lines and the 95% confidence intervals by shaded areas.

An overview of the study design was illustrated in Figure 1. The characteristics of UK Biobank participants included in this study were shown in Table S1. In this prospective study, there were 205,029 males and 241,750 females, and the mean age of participants was 56.75 years (range, 38–79 years). It showed that 2.22% of participants suffered fractures after 7.96 years of follow up. In the Cox regression analysis taking continuous BMI as exposure, BMI played a protective role for fracture in the basic model (model 0, adjusted for confounders such as age, sex, smoking status, drinking status, regular physical activity, the use of glucorticoid, socioeconomic status [SES], and processed meat intake, HR = 0.99, p = 0.0011). However, in restricted cubic spline analysis, a U-shape association was observed between BMI and fracture risk in model 0 (p < 0.0001 for nonlinearity), and the participants with BMI falling in the range of overweight (25.0 kg/m^2^–29.9 kg/m^2^) had the lowest risk to fracture (Figures 2A and S1). Compared with the participants who were overweight, the participants with underweight had increased risk of fracture in model 0 (hazard ratio [HR] = 1.54, 95% confidence interval [CI] 1.17 to 2.02, p = 0.0020) (Table 1) and model 1 (model 0 + falls) (HR = 1.57, 95% CI 1.19 to 2.06, p = 0.0013). Interestingly, the effect of underweight on fracture risk was attenuated by additionally adjusting for BMD (model 2 and model 3) (Table 1). Normal weight showed similar trends of effect as underweight. As for obesity, the risk effect of obesity on fracture was not significant in model 0 and model 1; however, when BMD was adjusted, the risk effect of obesity on fracture became larger with significance (p = 0.0222 in model 2) (Table 1). These results suggested that BMD might play important role in the pathway between BMI and fracture. In fact, we observed a positive linear correlation between BMI and BMD in our data (Figure S2A). We also performed observational analyses by excluding younger participants (i.e., <50 years old, N = 99,133) and found that the patterns of association in each model were similar to the aforementioned findings (Table S2). When stratified by gender, we observed that the effect of underweight/normal weight on fracture in females was smaller than in males (Table S3).

**Table 1:** Observational analyses for the relationship of obesity-related indices with fracture risk

Trait	Method	All			
		HR	95% CI lower	95% CI upper	P value
BMI	Model 0				
	Underweight	1.54	1.17	2.02	0.002
	Normal weight	1.16	1.10	1.23	2.12E-07
	Overweight	Ref	Ref	Ref	
	Obesity	1.03	0.97	1.10	0.3435
	Model 1				
	Underweight	1.57	1.19	2.06	0.0013
	Normal weight	1.18	1.11	1.25	1.54E-08
	Overweight	Ref	Ref	Ref	
	Obesity	1.00	0.94	1.07	0.896
	Model 2				
	Underweight	1.22	0.92	1.61	0.1681
	Normal weight	1.09	1.03	1.15	0.0036
	Overweight	Ref	Ref	Ref	
	Obesity	1.08	1.01	1.15	0.0222
	Model 3				
	Underweight	1.24	0.94	1.64	0.1328
	Normal weight	1.10	1.04	1.17	0.0007
	Overweight	Ref	Ref	Ref	
	Obesity	1.05	0.98	1.12	0.1344
WCadjBMI	Model 0	1.02	1.01	1.02	9.71E-16
	Model 1	1.02	1.01	1.02	4.68E-15
	Model 2	1.01	1.01	1.02	7.06E-10
	Model 3	1.01	1.01	1.02	2.09E-09
HCadjBMI	Model 0				
	HipadjBMI < 95 cm	1.10	0.96	1.26	0.1839
	HipadjBMI 95–105 cm	Ref	Ref	Ref	
	HipadjBMI ≥ 105 cm	1.09	1.03	1.14	0.0011
	Model 1				
	HipadjBMI < 95 cm	1.08	0.94	1.24	0.2881
	HipadjBMI 95–105 cm	Ref	Ref	Ref	
	HipadjBMI ≥ 105 cm	1.08	1.03	1.14	0.003
	Model 2				
	HipadjBMI < 95 cm	1.13	0.98	1.30	0.0821
	HipadjBMI 95–105 cm	Ref	Ref	Ref	
	HipadjBMI ≥ 105 cm	1.07	1.01	1.12	0.014
	Model 3				
	HipadjBMI < 95 cm	1.11	0.97	1.27	0.1442
	HipadjBMI 95–105 cm	Ref	Ref	Ref	
	HipadjBMI ≥ 105 cm	1.06	1.10	1.11	0.0265

Abbreviations: BMD, bone mineral density; BMI, body mass index; CI, confidence interval; HCadjBMI, hip circumference adjusted for BMI; HR, hazard ratio; WCadjBMI, waist circumference adjusted for BMI. Model 0 was adjusted for age, sex, smoking statue, alcohol drinker status, physical activity and the use of glucocorticoid, socioeconomic status and processed meat intake. Model 1 = model 0 + falls; model 2 = model 0 + BMD; model 3 = model 0 + falls + BMD.

Further, we conducted a series of mediation analyses to assess the role of BMD and falls on the observed association between BMI and fracture. Here, a suppression effect (Mackinnon et al., 2000) was observed because the direct effect and mediated effect had opposite direction (Table 2). In the basic model (model 0), the total effect of BMI on fracture was protective. When including BMD as the intermediary factor, the average direct effect (ADE) of BMI on fracture turned to risk with nonsignificance (p = 0.64), and the average causal mediation effect (ACME) by BMD was larger than the total effect of BMI (Table 2). These results, together with the results from Cox regression in different models, suggested that the protective effect of BMI on fracture was mainly mediated by BMD. In addition, only 13.8% of the intermediary effect of BMI on fracture was mediated by falls (Table 2).

**Table 2:** Assessment of the mediators (i.e. BMD and falls) for the association between obesity-related indices and fracture risk

Trait		BMD as mediator				Falls as mediator			
		Estimate	95% CI Lower	95% CI Upper	p-value	Estimate	95% CI Lower	95% CI Upper	p-value
BMI	Total Effect	−3.27E-04	−5.52E-04	0.00	<2.0E-16	−3.05E-04	−5.18E-04	0.00	<2.0E-16
	ACME (average)	−3.63E-04	−4.07E-04	0.00	<2.0E-16	4.18E-05	2.26E-05	0.00	<2.0E-16
	ADE (average)	3.64E-05	−1.45E-04	0.00	0.64	−3.47E-04	−5.53E-04	0.00	<2.0E-16
	Prop. Mediated (average)	1.12	0.74	2.26	<2.0E-16	−0.138	-0.26	−0.07	<2.0E-16
WCadjBMI	Total Effect	7.99E-05	6.73E-05	0.00	<2.0E-16	8.07E-05	7.12E-05	0.00	<2.0E-16
	ACME (average)	2.35E-05	1.60E-05	0.00	<2.0E-16	1.66E-06	−3.15E-06	0.00	0.56
	ADE (average)	5.64E-05	5.13E-05	0.00	<2.0E-16	7.91E-05	7.11E-05	0.00	<2.0E-16
	Prop. Mediated (average)	0.288	0.24	0.37	<2.0E-16	0.0208	−0.04	0.09	0.56
HCadjBMI	Total Effect	7.05E-05	5.71E-05	0.00	<2.0E-16	7.06E-05	6.06E-05	0.00	<2.0E-16
	ACME (average)	2.07E-05	1.21E-05	0.00	<2.0E-16	8.15E-07	-6.03E-06	0.00	0.80
	ADE (average)	4.98E-05	4.36E-05	0.00	<2.0E-16	6.98E-05	5.96E-05	0.00	<2.0E-16
	Prop. Mediated (average)	0.287	2.12E-01	0.41	<2.0E-16	0.0118	−9.31E-02	0.09	0.80

Abbreviations: ACME, average causal mediation effect; ADE, average direct effect; BMD, bone mineral density; BMI, body mass index; CI, confidence interval; HCadjBMI, hip circumference adjusted for BMI; WCadjBMI, waist circumference adjusted for BMI.

### The association of abdominal obesity indices with fracture

The restricted cubic spline analysis showed that there was a linear correlation between WCadjBMI and fracture risk (p = 0.2188 for nonlinearity) (Figure 2B). We found that WCadjBMI could increase the fracture risk in pooled samples in model 0 (HR = 1.02, 95% CI 1.01 to 1.02, p = 9.71E-16) (Table 1) and in both men and women (Table S3). The effect size of WCadjBMI on fracture did not change much when falls (model 1) and BMD (model 2) were further adjusted (Table 1). Based on the fully adjusted model (model 3), WCadjBMI was associated with incident fracture with a 1.0% higher risk (HR = 1.01, 95% CI 1.01–1.02, p = 2.09E-09). Besides, it was found that the higher WCadjBMI was related to lower BMD in our study (Figure S2B). We also observed similar findings between WCadjBMI and fracture risk when participants younger than 50 years were excluded (Table S2). The mediation analyses showed that 28.80% and 2.08% of the intermediary effect of WCadjBMI on fracture were mediated by BMD and falls (Table 2).

A J-shape association between HCadjBMI and fracture risk was observed in model 0 (p = 0.0178 for nonlinearity) (Figure 2C). Compared with those with 95–105 cm of HCadjBMI, the participants with smaller HCadjBMI (<95 cm) would not increase risk of fracture in all models (all p > 0.05) (Table 2), but the participants with larger HCadjBMI (≥105 cm) had increased risk of fracture in model 0 (HR = 1.09, 95% CI 1.03 to 1.14, p = 0.0011) (Table 1). Further adjusting for falls (model 1) and BMD (model 2) did not really attenuate the estimated HR for the association between HCadjBMI and fracture risk (Table 1). And, it was observed that the participants with higher HCadjBMI had lower BMD in our data (Figure S2C). Moreover, the relationship between HCadjBMI and fracture risk remained the same when participants younger than 50 years were excluded (Table S2). Similar trends were observed in the stratified analysis by sex (Table S3). For HCadjBMI, the intermediary effect by BMD and falls were 28.70% and 1.18%, respectively (Table 2).

### Genome-wide association study and the association of genetically determined obesity indices with fracture

In order to test the association between genetically determined obesity indices and fracture, we performed genome-wide association analyses for BMI, HCadjBMI, and WCadjBMI in 377,635 UKB participants of European ancestry. A total of 4,105,386 SNPs with MAF >0.05 were tested in the GWAS analyses, and we identified 54,134; 47,918; and 27,257 genome-wide significance (GWS) variants for BMI, HCadjBMI, and WCadjBMI (p < 5.0E-08), respectively. The Manhattan plots and QQ-plots for these traits were presented in Figures S3–S5. Finally, 1,456; 1,391; and 1,331 independent loci were identified for BMI, HCadjBMI, and WCadjBMI at genome-wide significance. To calculate the weighted genetic risk score (wGRS), we used independent SNPs with a p value less than 5.0 × E-06; therefore, we finally included 2,241 SNPs for BMI, 2,100 SNPs for HCadjBMI, and 1,510 SNPs for WCadjBMI in the wGRS calculation.

**Figure 3:**
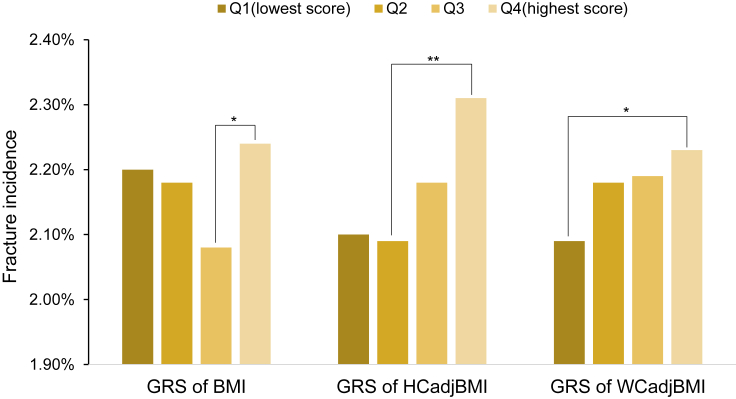
Bar charts illustrating the fracture incidence in the wGRS of BMI, HCadjBMI, and WCadjBMI by wGRS quartiles BMI, body mass index; HCadjBMI, hip circumference adjusted for BMI; WCadjBMI, waist circumference adjusted for BMI; wGRS, weighted Genetic Risk Score. Data are represented as fracture incidence. Significant differences are denoted above each plot (Chi-Square test; ∗p < 0.05; ∗∗p < 0.01).

We generated the wGRS of BMI/HCadjBMI/WCadjBMI for each individual, then we divided the wGRS value into four quartiles in the population. As shown in Figure 3, the incident of fracture was at the lowest in the Q3 group of BMI wGRS (incidence:2.08%), where the incident of fracture was higher in other three quartiles (Q1 2.20%, Q2 2.18%, Q4 2.24%), with the highest in the Q4 group (Figure 3); the difference between Q3 and Q4 was significant (p = 0.0304). As for WCadjBMI, the incident of fracture increased as the genetic risk score increased, with the lowest incident of fracture at Q1 (2.09%) and the highest incident of fracture at Q4 (2.31%), and the difference between them was significant (p = 0.0294). The lowest incident of fracture was observed in Q2 of HCadjBMI wGRS (2.09%), and we found statistically significant difference of fracture incident with the highest Q4 (2.31%) (p = 0.001) (Figure 3).

**Figure 4:**
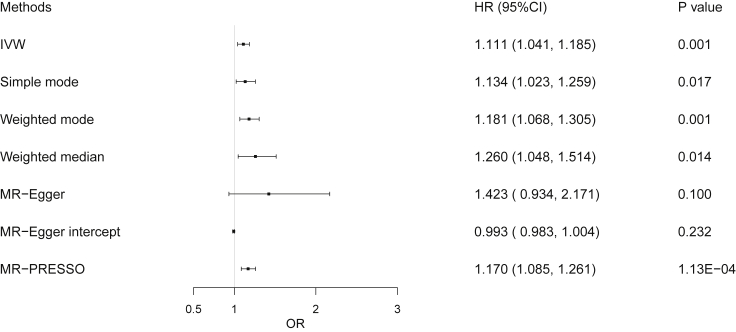
Associations between WCadjBMI and fracture in Mendelian randomization analyses Black dots in plot indicate hazard ratios (HR) and black lines in plot indicate 95% CI. IVW, inverse-variance weighted; MR-Egger, Mendelian randomization-egger; MR-PRESSO, Mendelian randomization pleiotropy residual sum and outlier; CI, confidence interval; WCadjBMI, waist circumference adjusted for BMI; OR, odds ratio.

Finally, as the WCadjBMI had linear correlation with fracture risk, we also performed two-sample MR analysis to assess the causal effect of WCadjBMI (76 SNPs selected, Table S4) on fracture. Similar to the observational analysis, it was found that a higher WCadjBMI was associated with higher fracture risk in two-sample MR analysis (IVW: odds ratio [OR] = 1.111, 95% CI 1.041 to 1.185, p = 0.001; MR-PRESSO: OR = 1.170, 95% CI 1.085 to 1.261, p = 1.13 × E-04) (Figure 4 and Table S5). Taking into account the heterogeneity in the analysis (p < 0.0001), the results from the simple model (OR = 1.134, p = 0.017), weighted model (OR = 1.181, p = 0.001), and weighted median (OR = 1.260, p = 0.014) were consistent with the IVW results (Figure 4 and Table S5).

## Discussion

In this prospective observational study, the lowest risk of fracture was observed within the overweight participants (25.0 kg/m^2^–29.9 kg/m^2^), the obesity individuals had higher risk of fracture when BMD was adjusted, and the protective effect of moderate-high BMI on fracture was mostly mediated by high BMD. However, the higher WCadjBMI was found to be related to higher risk of fracture, and only 28.80% of the effect was mediated by BMD. In our study, we observed J-shape for HCadjBMI and fracture risk. The BMD had a larger intermediary effect than falls in both general and abdominal obesity indices. By leveraging the genetic instrumental variables, the wGRS analysis provided additional evidences to support the aforementioned findings.

The interaction of obesity with fracture is complex and not as yet fully elucidated, and the effect of fat on the skeleton was mediated by both mechanical and biochemical factors (Gkastaris et al., 2020). Earlier studies reported that obesity, as demonstrated by high BMI, was protective against fragility fracture (Joakimsen et al., 1998; Kanis et al., 1999). In our study, we observed that moderate-high BMI was a protective factor for fracture; this is consistent with a recent large-scale meta-analysis of observational studies (Zhang et al., 2021b). Bone formation is stimulated by the weight-bearing effect caused by increased mechanical loading and higher fat padding as a result of elevated fat mass (Imai et al., 2010; Zhang et al., 2016; Savvidis et al., 2018). In our study, a significant positive relationship was also observed between the BMI and BMD measurements, which might explain why overweight (moderate-high BMI) had positive effect on fracture risk in our study.

In obese individuals, it would increase the risk of fracture when BMD was adjusted, probably because the BMD benefit from obesity would be insufficient to compensate for other risk factors. Previous studies had found that inflammation, which was more prevalent in obesity, had deleterious effects on bone strength and fracture risk (Ishii et al., 2013). Another factor involved was vitamin D deficiency, a very common situation among obese individuals that might have significant implications for skeletal health. Serum 25(OH)D concentrations were approximately 20% lower in obese people compared with those of normal weight (Ardawi et al., 2011; Walsh et al., 2016). Increased bone marrow fat in obesity might also have deleterious effects on bone (Biver et al., 2011). In addition, the mediation analysis suggested that BMD had larger intermediary effect than falls between BMI and fracture risk. Interestingly, our previous study to investigate the relationship between insomnia and fracture suggested a larger intermediary effect by falls than BMD (Qian et al., 2021).

Unlike general obesity, our study found that abdominal obesity (higher waist circumference) was associated with fracture risk in linear model. This relation might be explained by the effects of abdominal-obesity-related inflammation (Zhang et al., 2016; Gkastaris et al., 2020). Earlier studies demonstrated that inflammatory cytokines (including interleukin-1 [IL-1], IL-6, resistin, and tumor necrosis factor alpha [TNF-α]) that are released by visceral adipose tissue (VAT) would uncouple bone remodeling by suppressing bone formation and enhancing bone reabsorption (Kawai et al., 2012). These impulses decreased osteoblast differentiation and increased osteoclast recruitment, thereby uncoupling the bone remodeling unit (Rolland et al., 2012). It had been shown that abdominal obesity, compared with general obesity, was associated with higher levels of inflammatory markers; however, heavier weight that led to increased strain on bone could decrease the effect of inflammation on bone in individuals with general obesity (Pannacciulli et al., 2001, Stepanikova et al., 2017). In addition, it has been shown that higher high-sensitivity C-reactive protein levels were associated with a lower trabecular density, lower trabecular number, higher trabecular spacing, and more heterogeneous trabecular distribution (Kim et al., 2016). Abdominal obesity–relayed inflammation, therefore, could adversely influence trabecular bone score and bone quality index (Yamaguchi et al., 2009). In fact, we found that higher WCadjBMI was associated with lower BMD in our study. In addition, our findings suggested a J-shape relationship between HCadjBMI and fracture risk. In other words, the trend of the association between lower HCadjBMI and fracture risk might be mild, but higher HCadjBMI was significantly associated with fracture risk (p < 0.05 in all models). Previous study showed that hip circumference could represent adiposity of the hip region and larger hip circumference likely indicated greater subcutaneous fat accumulation, and finally leading to obesity-related inflammation (Hughes et al., 2004).

Furthermore, by using wGRS, it suggested that the incident of fracture was at the lowest in the Q3 group of BMI wGRS, where the incident of fracture was higher in other three quartiles, with the highest in the Q4 group. As for WCadjBMI, the incident of fracture increased, as the genetic risk score increased, with the lowest incident of fracture at Q1 and the highest incident of fracture at Q4. These findings were supportive to our observational results.

In summary, the observational and genetic evidence suggested that general and abdominal obesity operate differently as risk factors of fracture risk in old adults. A moderate-high BMI was a protective factor for fracture, and the BMD was the main intermediate factor. For abdominal obesity, higher WCadjBMI and HCadjBMI associated the higher risk of fracture, and BMD only mediated less than 30% of the effect, whereas falls had barely intermediate effect. Keeping moderate-high BMI would be of benefit to old people in terms of fracture risk; however, abdominal adiposity might increase risk of fracture; this is a compensation between mechanical and biochemical factors.

### Limitations of the study

Nonetheless, our study also has some limitations, some of which we have discussed. First, the participants in this study were of European descent; therefore, our findings might not apply to populations of other descents. Second, to enlarge the sample size and statistic power, we only evaluated the relationship between obesity-related indices and any-type fracture rather than fracture at specific anatomical site (such as hip and spine).

## STAR★Methods

### Key resources table

**Table:** 

REAGENT or RESOURCE	SOURCE	IDENTIFIER
**Software and algorithms**		

STATA 14.1 software	Stata Corporation, College Station, T	https://www.stata.com/
R version 4.0.3	Yavorska and Burgess, 2017; (Verbanck et al., 2018	https://www.r-project.org/
PLINK software	Chang et al., 2015	http://www.coggenomics.org/plink2

**Other**		

The phenotype and genotype data used in UK Biobank (Application 41376)	UK Biobank	https://www.ukbiobank.ac.uk/
The genotype data used in the GEnetic Factors for OSteoporosis Consortium (GEFOS)	GEFOS Consortium	http://www.gefos.org/

### Resource availability

#### Lead contact

Further information and requests for resources should be directed to and will be fulfilled by the Lead Contact, Hou-Feng Zheng (zhenghoufeng@westlake.edu.cn).

#### Materials availability

NA.

### Experimental model and subject details

The study participants in the present study were from UK Biobank. Ethics approval for the UK Biobank was obtained from the North West Multi-centre Research Ethics Committee (MREC) (Reference number 11/NW/0382). All UK Biobank participants were recruited with fully informed consent. The prospective study included 456,921 participants (205,029 males and 241,750 females) at baseline and the age of participants ranged from 38 to 79 years-old, with the mean age of 56.75 years-old (Table S1). We observed that 10,142 participants (2,883 males and 7,259 females) developed an incident fracture with a median of 7.96 years of follow-up. In the multivariable Cox regression, the basic model was adjusted for confounders, including age, sex, smoking status, alcohol status, physical activity, the use of glucorticoid, Socioeconomic Status (SES) and processed meat intake, to control the influence of these factors on the association between obesity and fracture risk. Analyses stratified by sex were also performed. The detailed analysis models could be found in the Method details under “Observational study” section.

### Method details

#### Data sources and study participants

An overview of the study design was illustrated in Figure 1. The phenotype and genotype data used in the present study were from UK Biobank (Application 41376) (Bai et al., 2020), which comprises ∼500,000 individuals recruiting between 2006 and 2010 from primary care practices across UK. In this study, we excluded 30,487 non-European participants to minimize the population stratification bias. Then, we excluded participants with secondary fractures and those with fracture history (N = 15,011). Therefore, in the prospective study, there were 456,921 participants (205,029 males and 241,750 females) at baseline and 10,142 participants developed an incident fracture during a median of 7.96 years of follow-up. Among these participants, there were 454,980, 454,885 and 454,896 participants with BMI, WCadjBMI and HCadjBMI, respectively. The endpoint was the first diagnosis of any-type fracture, excluding fracture at skull, face, hands, and feet, pathological fractures, atypical femoral fractures and periprosthetic fractures. These cases were from either questionnaire-based self-reported fractures or hospital-based fractures using the ICD-9 or ICD-10 codes (Table S6).

The BMI was derived using height (measured in whole centimeters) and weight (to the nearest 0.1 kg) measured at baseline in the UK Biobank. BMI was divided into underweight (<18.5 kg/m^2^), normal weight (18.5 kg/m^2^-24.9 kg/m^2^), overweight (25.0 kg/m^2^-29.9 kg/m^2^) and obesity (≥30.0 kg/m^2^) based on World Health Organization guidelines (Low et al., 2009). To generate the measurement of waist circumference adjusted for BMI (WCadjBMI), we first performed linear regression for WC with independent variable BMI, and the residual of WC for each individual was predicted. Then the WCadjBMI of each individual was calculated as the sum of the mean of WC and the residual. The hip circumference adjusted for BMI (HCadjBMI) was derived in the same way.

In order to assess Socioeconomic Status (SES), average total household income before tax (Field ID: 738), education score (England) (Field ID: 26414) and current employment statue (Field ID: 6142) were used to measure SES according to previous studies (Tyrrell et al., 2016; Zhang et al., 2021a). For average total household income before tax, we defined the option from less than £18,000 as low (0 score), £18,000-£30,999, £31,000-£51,999 and £52,000-£100,000 as medium (1 score), and >£100,000 as high (2 score). For education score (England), we divided it into three levels (low, 0 score; medium, 1 score; and high, 2 score) with consideration of practical interpretation and sample size within levels. As UK Biobank only acquired employment status instead of information on specific occupation at baseline, therefore we regrouped participants into two groups: unemployed (0 score) and employed (1 score, including those in paid employment or self-employed, retired, doing unpaid or voluntary work, or being full or part time students) (Raisi-Estabragh et al., 2020). SES score was calculated as the sum of all these component scores (0–5 scores) and was divided into three levels (low SES, participants who have SES score 0 to 1; medium SES, participants who have SES score 2 to 3; and high SES, participants who have SES score 4 to 5).

Processed meat intake (Field ID:1349) was obtained through questionnaires in UK Biobank, and we considered it as a marker of poor diet quality (Raisi-Estabragh et al., 2020). Processed meat intake was coded as never, less than once a week, once a week, 2–4 times a week, 5–6 times a week, once or more daily, do not know, and prefer not to answer. We defined the option from never as never (0 score), less than once a week and once a week as occasionally (1 score), and 2–4 times a week, 5–6 times a week and once or more daily as often (2 score).

#### Observational study

In the prospective study of fracture risk, follow-up time was calculated from the date of attending the UK Biobank to the diagnosis of fracture, death, or the censoring date (31 March 2017). We then investigated association between the obesity indices (BMI, WCadjBMI, and HCadjBMI) and the risk of fracture using Cox regression. Restricted cubic spline was used to model the potential non-linear association of obesity-related traits with fracture risk. In the multivariable Cox regression, the basic model was adjusted for confounders, including age, sex, smoking status, alcohol status, physical activity, the use of glucorticoid, Socioeconomic Status (SES) and processed meat intake (model 0). Additional covariables such as falls and BMD were also included to set different models (model 1 = model 0 + falls, model 2 = model 0 + BMD and model 3 = model 0 + BMD + falls). Detailed information on these covariates is provided in Table S7.

Furthermore, we performed mediation analysis to explore whether the relationship between obesity-related indices (exposure) and fracture (outcome) could be explained, at least partially, by an intermediate variable (mediator). Here we set the BMD and falls as the mediators from the prior knowledge (Trajanoska and Rivadeneira, 2019; Ganz and Latham, 2020). We applied the causal mediation analysis method to dissect the total effect of exposure into direct and indirect effect, and to examine the indirect effect which was transmitted via mediator to the outcome. The mediation analysis was performed using the R packages of ‘mediation’ and adjusting the age, sex, smoking status, alcohol consumption, physical activity and the use of glucorticoid, SES and processed meat intake.

#### Genome wide association study

We conducted genome wide association study for BMI, HCadjBMI and WCadjBMI within UK Biobank. In the association study, a total of 392,422 samples were retained after excluding individuals with inconsistent gender information (N = 372) and relatedness (N = 63,497). We further excluded samples with call rate less than 95% (377,635 sample left). Of the 377,635 individuals, 204,344 (54.11%) were females and 173,291 were males (45.89%). We then performed genotype QC, including minor allele frequency (MAF) > 0.05, genotype call rate >0.95, Hardy-Weinberg p > 1.0E-06 and info score (exclude the low quality imputed SNPs) > 0.7. Finally, 4,105,386 SNPs were left in the association analysis. The following covariates were included as fixed effects in model: age, sex, smoking, alcohol consumption, assessment center and ancestry informative principal components 1 to 5.

#### Weighted genetic risk score (wGRS) analysis

We calculated the weighted genetic risk score (wGRS) using the independent SNPs with p value less than 5.0E-06 for BMI, HCadjBMI and WCadjBMI within the UK Biobank dataset, respectively. The wGRS method formula was:

$$\text{wGRSi}=\sum_{i}^{N}\text{βi}\;\text{SNPi}$$

where βi was the effect estimate of the ith SNP derived from the GWAS of BMI, HCadjBMI and WCadjBMI in UK Biobank, N was the number of instrumental SNPs selected. Then we divided the wGRS value of BMI, HCadjBMI and WCadjBMI into 4 quartiles in the population. For BMI wGRS, there were 2,015 fractures and 89,701 controls in Q1 (lowest score), 1,996 fractures and 89,688 controls in Q2, 1,904 fractures and 89,783 controls in Q3 and 2,053 fractures and 89,684 controls in Q4 (highest score). For HCadjBMI wGRS, there were 1,931, 1,917, 2,000, 2,120 fractures and 89,896, 89,824, 89,667, 89,469 controls in Q1, Q2, Q3 and Q4, respectively. Similarly, there were 1,919, 2,001, 2,004, 2,048 and 89,859, 89,754, 89,637, 89,606 controls in Q1, Q2, Q3 and Q4 of WCadjBMI wGRS.

#### Two-sample MR

We performed two-sample summary-level MR analyses for WCadjBMI. 76 independent genetic variants associated with WCadjBMI at genome-wide significance level (p < 5.0E-08) (Table S4) were chose from Shungin D et al. (Shungin et al., 2015). Summary-level data for the outcome (fracture) were available from the previous published GWAS (Morris et al., 2019).

### Quantification and statistical analysis

For Observational study, all these statistical analyses were conducted in R version 4.0.3 and STATA 14.1 software. A Bonferroni-corrected threshold of p < 0.05 was considered statistically significant.

The GWAS analysis of BMI, WCadjBMI and HCadjBMI was performed using PLINK software (http://www.coggenomics.org/plink2), and we used the option --clump-r2 and --clump -kb to obtain the independent locus.

The wGRS method was performed using PLINK software (http://www.coggenomics.org/plink2) with the command of *--score sum* to obtain the sum of valid per-allele scores (Chang et al., 2015). The asterisks indicate significant differences between groups (∗ = p < 0.05; ∗∗ = p < 0.01). The different letters above each of plots indicate significant differences according to Chi-Square test. The Chi square test for quartile groups was conducted in STATA 14.1 software.

For Two-sample MR, all statistical analyses were performed with R 4.0.3. The IVW, simple mode, weighted mode, weighted-median, and MR-Egger methods were performed using the “MendelianRandomization” package (Yavorska and Burgess, 2017). The MR-PRESSO approach was performed using the “MR-PRESSO” package (Verbanck et al., 2018). The two-sided p value of less than 0.05 was considered statistically significant.

## Data Availability

This paper analyzes the existing and publicly available data, the accession IDs for any dataset are listed in the Key resources table. This paper does not report original code, any additional information required to reanalyze the data reported in this paper is available from the lead contact upon request.
