# Supplementary material for: General and abdominal obesity operate differently as influencing factors of fracture risk in old adults
Source: iScience. 2022 May 25;25(6):104466. doi: 10.1016/j.isci.2022.104466 (PMC9167983; doi:10.1016/j.isci.2022.104466)
Supplement: Document S1. Figures S1–S5 and Tables S1–S7 [file mmc1.pdf]

iScience, Volume *25*

Supplemental information

General and abdominal obesity operate differently as influencing factors of fracture risk in old adults

Xiao-Wei Zhu, Ke-Qi Liu, Cheng-Da Yuan, Jiang-Wei Xia, Yu Qian, Lin Xu, Jian-Hua Gao, Xiao-Li Rong, Guo-Bo Chen, David Karasik, Shu-Yang Xie, and Hou-Feng Zheng

Supplemental figures

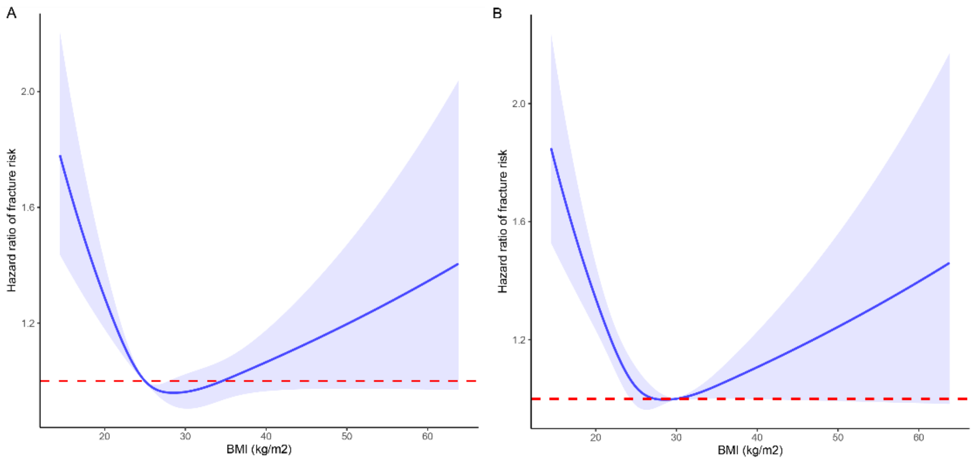

Figure S1. Observational association of BMI with fracture risk using a restricted cubic spline based on model 1 by different cut points, related to Figure 2. A) BMI = 25kg/m^2; B) BMI = 30kg/m^2. Hazard ratios are indicated by solid lines and the 95% confidence intervals by shaded areas. In all these analyses, models were adjusted for risk factors for fracture, including age, sex, smoking statue, alcohol drinker status, physical activity and the use of glucocorticoid, socioeconomic status and processed meat intake.

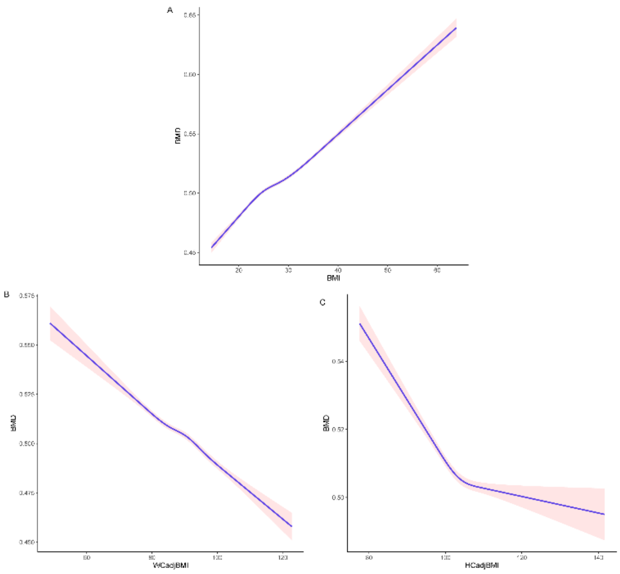

Figure S2. The relationship between BMI/WCadjBMI and BMD using a restricted cubic spline based on model 0, related to Table 1. A) BMI and BMD; B) WCadjBMI and BMD; C) HCadjBMI and BMD. Model 0 was adjusted for age, sex, smoking statue, alcohol drinker status, physical activity and the use of glucocorticoid, socioeconomic status and processed meat intake.

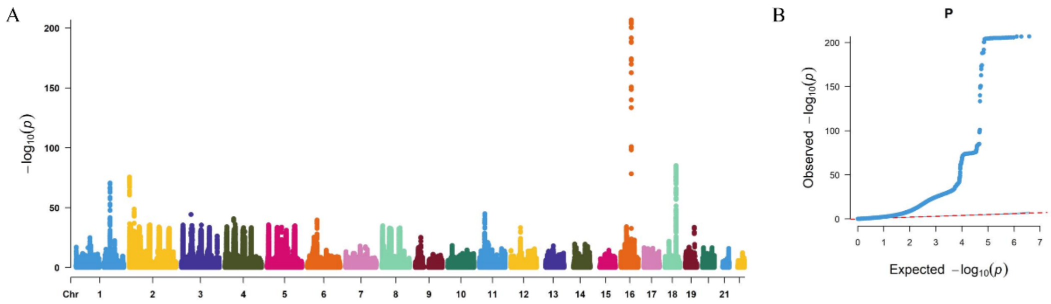

Figure S3. Genome-wide association of BMI graphed by chromosome positions and −log10 P-value (Manhattan plot), and quantile-quantile plot of all SNPs, related to Figure 3. A) Manhattan plot: The Y axis shows −log10 P-values, and the X axis shows chromosome positions. B) QQ-plot: The Y axis shows observed −log10 P-values, and the X axis shows the expected −log10 P-values. Each SNP is plotted as a blue dot, and the dash line indicates null hypothesis of no true association.

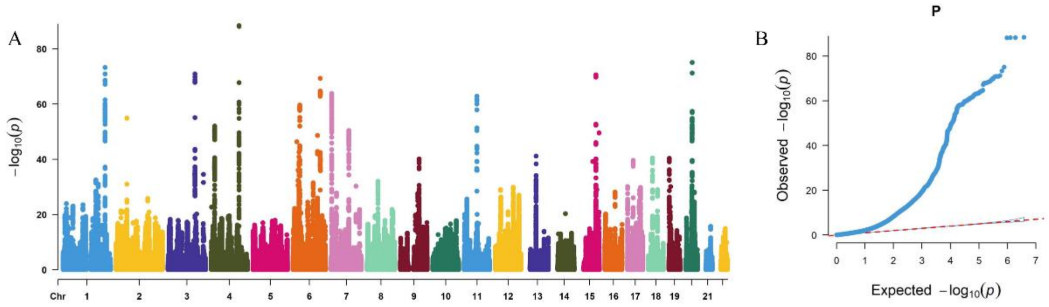

Figure S4. Genome-wide association of HCadjBMI graphed by chromosome positions and −log10 P-value (Manhattan plot), and quantile-quantile plot of all SNPs, related to Figure 3. A) Manhattan plot: The Y axis shows −log10 P-values, and the X axis shows chromosome positions. B) QQ-plot: The Y axis shows observed −log10 P-values, and the X axis shows the expected −log10 P-values. Each SNP is plotted as a blue dot, and the dash line indicates null hypothesis of no true association.

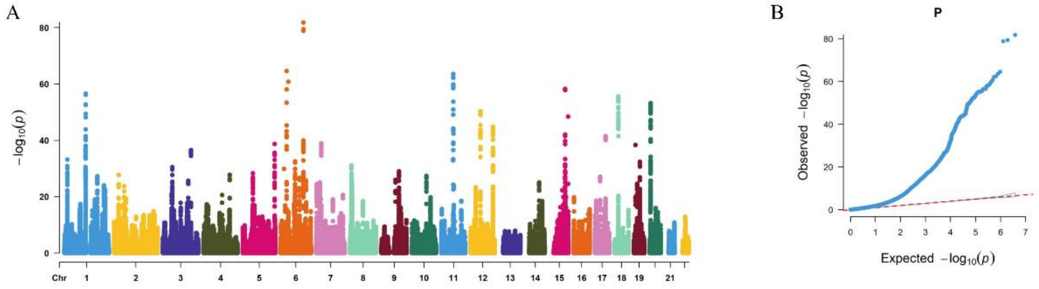

Figure S5. Genome-wide association of WCadjBMI graphed by chromosome positions and −log10 P-value (Manhattan plot), and quantile-quantile plot of all SNPs, related to Figure 3. A) Manhattan plot: The Y axis shows −log10 P-values, and the X axis shows chromosome positions. B) QQ-plot: The Y axis shows observed −log10 P-values, and the X axis shows the expected −log10 P-values. Each SNP is plotted as a blue dot, and the dash line indicates null hypothesis of no true association.

Supplemental tables

Table S1. Sample characteristics in UK Biobank participants included in this study and the numbers of the outcome events, related to Table 1.

Characteristic	Participants without fracture		Participants with fracture		P-value
	Sample size	Mean (SD)or %	Sample size	Mean (SD)or %	
Gender					
Males	205,029	45.89	2,883	28.43	
Females	241,750	54.11	7,259	71.57	
Age	446,779	56.70(8.04)	10,142	59.22（7.34）	<0.0001
BMI (kg/m^2)	444,936	27.41(4.78)	10,044	27.17（5.00）	<0.0001
WCadjBMI (cm)	444,845	90.30(7.84)	10,040	89.30（7.74）	<0.0001
HCad$_j$BMI (cm)	444,855	103.42（4.64）	10,041	103.80（4.84）	<0.0001
Alcohol drinker status					<0.0001
Never	14,438	3.23	457	4.52	
Previous	15,440	3.46	465	4.6	
Current	416,516	93.31	9,195	90.89	
Smoking status					<0.0001
Never	240,409	54.00	5,351	53.02	
Previous	158,425	35.58	3,557	35.25	
Current	46,379	10.42	1,184	11.73	
IPAQ activity group					0.068

Low	66,583	19.58	2,053	19.36	
Moderate	145,832	40.98	4,103	38.68	
High	144,376	39.44	4,451	41.96	
Falls					<0.0001
No falls	359,962	81	7,110	70.3	
Falls	85956	19.28	3,004	29.7	
The use of glucorticoid					0.443
Yes	381,432	88.11	8,975	88.36	
No	51,456	11.89	1,182	11.64	
SES					<0.0001
Low	14,942	3.71	418	5.09	
Medium	275,484	68.38	5,937	72.24	
High	112,454	27.91	1,864	22.68	
Processed meat intake					<0.0001
Never	37,140	8.59	1,097	10.83	
Occasionally	259,245	59.98	6,189	61.11	
Often	135,842	31.43	2,841	28.05	

Abbreviations: BMI, body mineral density; HCadjBMI, hip circumference adjusted for BMI; IPAQ, international physical activity questionnaire; SES, Socioeconomic Status; WCadjBMI, waist circumference adjusted for BMI.

Table S2. Observational analyses for the relationships of obesity-related traits with fracture risk among aged 50 years or older, related to Table 1.

Trait	Method	HR	All		
			95%CI lower	95%CI upper	P value
BMI	Model 0				
	Underweight	1.52	1.13	2.06	0.0059
	Normal weight	1.18	1.11	1.26	5.40E-08
	Overweight	Ref	Ref	Ref	
	Obesity	1.03	0.96	1.11	0.3594
	Model 1				
	Underweight	1.56	1.15	2.1	0.0037
	Normal weight	1.20	1.13	1.28	5.02E-09
	Overweight	Ref	Ref	Ref	
	Obesity	1.01	0.94	1.08	0.8662
	Model 2				
	Underweight	1.19	0.87	1.62	0.268
	Normal weight	1.11	1.04	1.18	0.0015
	Overweight	Ref	Ref	Ref	
	Obesity	1.09	1.01	1.17	0.0211
	Model 3				
	Underweight	1.22	0.9	1.66	0.21
	Normal weight	1.12	1.05	1.19	0.0003
	Overweight	Ref	Ref	Ref	
	Obesity	1.06	0.99	1.14	0.114
WCadjBMI	Model 0	1.02	1.02	1.02	< 2.00E-16
	Model 1	1.02	1.02	1.02	< 2.00E-16

	Model 2	1.02	1.01	1.02	2.19E-12
	Model 3	1.02	1.01	1.02	9.09E-12
HCadjBMI	Model 0				
	HipadjBMI < 95 cm	1.03	0.88	1.2	0.7239
	HipadjBMI 95-105 cm	Ref	Ref	Ref	
	HipadjBMI >=105 cm	1.09	1.03	1.15	0.0018
	Model 1				
	HipadjBMI < 95 cm	1.01	0.87	1.17	0.9187
	HipadjBMI 95-105 cm	Ref	Ref	Ref	
	HipadjBMI >=105 cm	1.08	1.02	1.14	0.0046
	Model 2				
	HipadjBMI < 95 cm	1.06	0.91	1.23	0.4848
	HipadjBMI 95-105 cm	Ref	Ref	Ref	
	HipadjBMI >=105 cm	1.07	1.01	1.13	0.0132
	Model 3				
	HipadjBMI < 95 cm	1.03	0.89	1.21	0.6686
	HipadjBMI 95-105 cm	Ref	Ref	Ref	
	HipadjBMI >=105 cm	1.07	1.01	1.13	0.0245

Abbreviations: BMD, bone mineral density; BMI, body mineral density; CI, confidence interval; HCadjBMI, hip circumference adjusted for BMI; HR, hazard ratio; WCadjBMI, waist circumference adjusted for BMI. Model 0 was adjusted for age, sex, smoking statue, alcohol drinker status, physical activity and the use of glucocorticoid, socioeconomic status and processed meat intake. Model 1 = Model 0 + falls; Model 2 = Model 0 + BMD; Model 3 = Model 0+ falls + BMD.

Table S3. Observational analyses for the relationships of obesity-related traits with fracture risk in male and female, related to Table 1.

Trait	Method	Male				Female			
		HR	95%CI lower	95%CI upper	P value	HR	95%CI lower	95%CI upper	P value
BMI	Model 0								
	Underweight	2.59	1.39	4.84	0.0028	1.43	1.05	1.93	0.0222
	Normal weight	1.29	1.16	1.43	1.21E-06	1.12	1.05	1.20	6.17E-04
	Overweight	Ref	Ref	Ref		Ref	Ref	Ref	
	Obesity	1.12	1.01	1.25	0.0352	0.99	0.91	1.07	0.777
	Model 1								
	Underweight	2.46	1.32	4.60	0.0047	1.45	1.07	1.97	0.0158
	Normal weight	1.30	1.17	1.44	8.31E-07	1.14	1.06	1.22	1.51E-04
	Overweight	Ref	Ref	Ref		Ref	Ref	Ref	
	Obesity	1.09	0.98	1.22	0.1033	0.96	0.89	1.04	0.3604
	Model 2								
	Underweight	1.81	0.97	3.40	0.0629	1.13	0.82	1.54	0.4543
	Normal weight	1.19	1.07	1.32	0.0011	1.06	0.99	1.13	0.1108
	Overweight	Ref	Ref	Ref		Ref	Ref	Ref	
	Obesity	1.15	1.03	1.25	0.0105	1.06	0.97	1.15	0.1913
	Model 3								
	Underweight	1.73	0.92	3.23	0.0884	1.15	0.84	1.57	0.3803
	Normal weight	1.19	1.08	1.32	0.0009	1.07	1.00	1.15	0.0498
	Overweight	Ref	Ref	Ref		Ref	Ref	Ref	
	Obesity	1.12	1.01	1.25	0.0381	1.03	0.95	1.12	0.4785
WCadjBMI	Model 0	1.02	1.01	1.03	4.32E-06	1.02	1.01	1.02	1.72E-11

	Model 1	1.02	1.01	1.03	2.10E-05	1.02	1.01	1.02	2.92E-11
	Model 2	1.01	1.01	1.02	9.95E-04	1.01	1.01	1.02	6.20E-08
	Model 3	1.01	1.00	1.02	0.0027	1.01	1.01	1.02	9.52E-08
HCadjBMI	Model 0								
	HipadjBMI < 95 cm	1.23	1.00	1.52	0.0482	1.01	0.84	1.21	0.9323
	HipadjBMI 95-105 cm	Ref	Ref	Ref		Ref	Ref	Ref	
	HipadjBMI >=105 cm	1.13	1.03	1.23	0.0123	1.08	1.02	1.15	0.0135
	Model 1								
	HipadjBMI < 95 cm	1.19	0.97	1.47	0.1014	1.00	0.83	1.20	0.9871
	HipadjBMI 95-105 cm	Ref	Ref	Ref		Ref	Ref	Ref	
	HipadjBMI >=105 cm	1.12	1.02	1.23	0.0153	1.00	1.01	1.14	0.0269
	Model 2								
	HipadjBMI < 95 cm	1.26	1.02	1.55	0.034	1.06	0.88	1.27	0.5556
	HipadjBMI 95-105 cm	Ref	Ref	Ref		Ref	Ref	Ref	
	HipadjBMI >=105 cm	1.09	0.99	1.19	0.082	1.07	1.00	1.13	0.0409
	Model 3								
	HipadjBMI < 95 cm	1.21	0.98	1.49	0.0791	1.05	0.87	1.26	0.6258
	HipadjBMI 95-105 cm	Ref	Ref	Ref		Ref	Ref	Ref	
	HipadjBMI >=105 cm	1.08	0.99	1.19	0.093	1.06	1.00	1.13	0.0661

Abbreviations: BMD, bone mineral density; BMI, body mineral density; CI, confidence interval; HCadjBMI, hip circumference adjusted for BMI; HR, hazard ratio; WCadjBMI, waist circumference adjusted for BMI. Model 0 was adjusted for age, sex, smoking statue, alcohol drinker status, physical activity and the use of glucocorticoid, socioeconomic status and processed meat intake. Model 1 = Model 0 + falls; Model2 = Model 0 + BMD; Model 3 = Model 0 + falls + BMD.

Table S4. The characteristics of the associations of instrumental variables of WCadjBMI with fracture risk for Two-sample MR, related to Figure 4.

SNP	WCadjBMI			
	Effective allele	Beta	SE	P-value
rs7536458	T	0.0305	0.0038	1.24E-15
rs10923712	A	0.0354	0.0035	1.07E-24
rs11205277	A	-0.0270	0.0036	1.34E-13
rs9435732	T	-0.0309	0.0038	4.12E-16
rs2274432	A	0.0251	0.0036	1.75E-12
rs12048049	C	-0.0256	0.0037	2.45E-12
rs3897379	A	0.0160	0.0043	2.12E-04
rs12127195	A	0.0211	0.0037	7.67E-09
rs10925060	T	0.0174	0.0041	2.20E-05
rs3862030	A	0.0206	0.0033	5.83E-10
rs1784203	A	0.0307	0.0054	1.31E-08
rs12317176	T	0.0205	0.0035	5.88E-09
rs2638953	C	0.0237	0.0036	6.53E-11
rs2071449	A	0.0315	0.0036	2.47E-18
rs7970350	T	-0.0188	0.0034	3.76E-08
rs2160077	A	-0.0181	0.0033	4.46E-08
rs4246302	A	-0.0216	0.0037	5.73E-09
rs7166081	A	0.0236	0.0039	2.12E-09
rs4886782	A	-0.0245	0.0036	5.98E-12
rs7162542	C	-0.0379	0.0034	9.75E-29
rs1879529	T	-0.0239	0.0038	2.86E-10
rs2047937	T	-0.0186	0.0034	4.67E-08
rs16957304	A	0.0591	0.0106	2.47E-08
rs3760318	A	-0.0213	0.0035	9.05E-10
rs882367	T	-0.0267	0.0036	1.09E-13
rs882367	T	-0.0267	0.0036	1.09E-13
rs4239436	A	-0.0405	0.0041	1.01E-22
rs12608504	A	0.0201	0.0036	1.48E-08
rs3786897	A	-0.0199	0.0035	8.77E-09
rs4542783	T	0.0227	0.0040	1.69E-08
rs2124969	T	-0.0199	0.0034	7.06E-09
rs10195252	T	0.0114	0.0034	9.44E-04
rs6437061	A	0.0166	0.0035	1.77E-06
rs12991495	T	0.0281	0.0037	6.18E-14
rs6715793	T	0.0193	0.0034	1.44E-08
rs3791679	A	0.0353	0.0039	2.06E-19
rs2052670	A	-0.0200	0.0035	1.53E-08
rs1884897	A	0.0320	0.0035	6.56E-20
rs9977276	T	-0.0218	0.0040	4.37E-08

rs2294239	A	0.0191	0.0035	3.49E-08
rs6772896	T	0.0242	0.0036	1.79E-11
rs7621331	A	0.0207	0.0036	9.41E-09
rs1344674	A	-0.0240	0.0033	4.33E-13
rs17451107	T	0.0265	0.0036	1.25E-13
rs12493901	A	-0.0209	0.0034	8.28E-10
rs12489828	T	-0.0197	0.0034	1.03E-08
rs9860730	A	0.0219	0.0037	2.07E-09
rs12330322	T	-0.0223	0.0040	3.15E-08
rs1812175	A	-0.0330	0.0045	3.95E-13
rs7684221	A	-0.0259	0.0047	4.15E-08
rs2197271	C	-0.0287	0.0039	2.17E-13
rs10041657	A	0.0251	0.0040	2.88E-10
rs272869	A	-0.0212	0.0034	6.68E-10
rs17472426	T	0.0144	0.0067	3.11E-02
rs4868125	C	-0.0215	0.0036	2.93E-09
rs10516107	A	0.0232	0.0036	8.29E-11
rs6556301	T	0.0278	0.0039	1.82E-12
rs7733331	T	0.0213	0.0034	5.74E-10
rs13173241	A	0.0259	0.0043	1.65E-09
rs395962	T	0.0288	0.0036	1.33E-15
rs2745353	T	0.0293	0.0033	7.88E-19
rs6570507	A	-0.0245	0.0037	5.73E-11
rs7773004	A	0.0263	0.0033	1.93E-15
rs1776897	T	-0.0613	0.0067	5.58E-20
rs13210323	A	0.0216	0.0038	1.45E-08
rs998584	A	0.0293	0.0038	6.45E-15
rs12207675	T	-0.0308	0.0052	3.14E-09
rs822531	T	0.0244	0.0044	3.71E-08
rs2214442	A	-0.0265	0.0045	3.89E-09
rs4141278	T	-0.0335	0.0043	3.39E-15
rs7801581	T	0.0271	0.0042	8.01E-11
rs798502	A	0.0243	0.0037	2.95E-11
rs849140	T	0.0288	0.0034	4.74E-17
rs7830933	A	0.0174	0.0039	9.01E-06
rs12679556	T	-0.0263	0.0039	1.26E-11
rs11144688	A	-0.0336	0.0060	1.87E-08
rs473902	T	0.0490	0.0071	4.35E-12

Table S5. The associations of genetic determined WCadjBMI with fracture risk by using different MR methods, related to Figure 4.

Traits	OR	95%CI	P	Q' P value
WCadjBMI→Fracture				
IVW	1.111	1.041 to 1.185	0.001	<0.0001
Simple mode	1.134	1.023 to 1.259	0.017	
Weighted mode	1.181	1.068 to 1.305	0.001	
Weighted median	1.26	1.048 to 1.514	0.014	
MR-Egger	1.423	0.934 to 2.171	0.1	
MR-Egger intercept	0.993	0.983 to 1.004	0.232	
MR-PRESSO*	1.17	1.085 to 1.261	1.13E-04	0.347

Abbreviations: CI, confidence interval; IVW, inverse-variance weighted; MR-Egger, Mendelian randomization-egger; MR-PRESSO, Mendelian randomization pleiotropy residual sum and outlier; OR, odds ratio; WCadjBMI, waist circumference adjusted for BMI; Q' p value: Test for Heterogeneity of IVW. *IV outliers detected: rs2745353, rs3862030, rs3897379, rs7536458.

Table S6. Detailed information on self-reported codes for the main obesity related index (BMI, waist circumference and hip circumference), the ICD-9 and ICD-10 codes for non-secondary fracture, and the codes for the corresponding diagnosis time in UK Biobank, related to Table 1.

Phenotype	Field ID	Code
Exposure		
BMI	21001	\
Waist circumference	48	\
Hip circumference	49	\
Outcome		
Fracture	20002	1634, 1635, 1636, 1637, 1638, 1639, 1640, 1644, 1645, 1646, 1648, 1649, 1650, 1651, 1653, 1654, 1655, 1656
	41203	73382, 73383, 73384, 73385, 73386, 73387, 805, 8050, 8052, 8054, 8056, 8058; 806, 8064, 807, 8070, 8072, 8074, 809, 8090, 8091, 812, 8120, 8121, 8122, 8123, 8124, 8125, 813, 8130, 8131, 8132, 8134, 8135, 814,8140, 8141, 815, 8150, 8151, 816, 8160, 8161, 817, 8170, 820, 8200, 8202, 8208, 821, 8210, 8211, 8212, 822, 8220, 8221; 823, 8230, 8231, 8232, 8233, 824, 8240, 8241, 8242, 8244, 8245, 8246, 8247, 8248, 8249, 825, 8250, 8252, 8253, 826, 8260, 8261, 9052, 9053, 9054

	41270	M484, M4840, M4842, M4845, M4846, M4847, M4848, M4849, M8402, M8403, M8404, M8406, M8407, M8412, M8413, M8414, M8416, M8417, M8422, M8423, M8424, M8426, M8427, M8436, M8437, S120, S1200, S121, S1210, S122, S1220, S127, S1270, S22, S220, S2200, S221, S2210, S222, S2220, S223, S2230, S2231, S224, S2240, S2241, S225, S2250, S2251, S228, S2280, S229, S2290, S32, S320, S3200, S323, S3230, S3231, S324, S3240, S3241, S422, S4220, S4221, S423, S4230, S4231, S424, S4240, S4241, S52, S520, S5200, S5201, S521, S5210, S5211, S522, S522, S5220, S5221, S523, S5230, S5231, S524, S5240, S5241, S525, S5250, S5251, S526, S5260, S5261, S527, S5270, S5271, S528, S5280, S5281, S529, S5290, S62, S620, S6200, S621, S6210, S6211, S622, S6220, S6221, S623, S6230, S6231, S624, S6240, S6241, S625, S6250, S6251, S626, S6260, S6261, S627, S6270, S6271, S628, S6280, S6281, S72, S720, S7200, S7201, S721, S7210, S7211, S722, S7220, S7221, S723, S72230, S7231, S724, S7240, S7241, S727, S7270, S728, S7280, S729, S7290, S7291, S82, S820, S8200, S8201, S821, S8210, S8211, S822, S8220, S8221, S823, S8231, S824, S8240, S8241, S825, S8250, S8251, S826, S8260, S8261, S827, S8270, S8271, S828, S8280, S8281, S8286, S829, S8290, S8291, S92, S920, S9200, S9201, S921, S9210, S9211, S922, S9220, S9221, S923, S9230, S9231, S924, S9240, S9241, S925, S9250, S9251, S927, S9270, S9271, S929, S9290, S9291, T022, T0220, T023, T0230, T0231, T024, T0240, T0241, T025, T0250, T0251, T026, T0260, T08, T08X0, T10, T10X0, T12, T911, T921, T922, T931, T932
Year when non-cancer illness first diagnosed	2008	
Date of first in-patient diagnosis based on ICD-9	41263	\
Date of first in-patient diagnosis based on ICD-10	41280	\

Table S7. Detailed information on the definition and self-reported UK Biobank field codes for covariates which were adjusted in observational studies, related to Table 1.

Covariates	Source and definition	Field ID
Age	Age at recruitment in UK Biobank.	21022
Alcohol consumption	UK Biobank questionnaire at baseline; the status of alcohol consumption was categorized as never, previous, or current.	20117
Bone mineral density	Physical measurement at baseline; the estimates of bone mineral density of the left calcaneus, which were measured by trained staffs using quantitative ultrasound.	4105
Falls	UK Biobank questionnaire at baseline; the status of falls was categorized as having more than one fall, only one fall, or not in the last year.	2296
Sex	UK Biobank questionnaire at baseline; the status of sex was categorized as male, or female.	31
Smoking status	UK Biobank questionnaire at baseline; the status of smoking was categorized as never, previous, or current smoking.	20116
Average total household income before tax	UK Biobank questionnaire at baseline; the status of average total household income before tax was divided into less than £18,000, £18,000-£30,999, £31 000-£51 999, £52,000-£100 000, >£100,000, do not know, or prefer not to answer.	738
Education score (England)	UK Biobank questionnaire at baseline.	26414
Current employment status	UK Biobank questionnaire at baseline. We regrouped participants into two groups: employed and unemployed.	6142

Processed meat intake	UK Biobank questionnaire at baseline.	1349
Physical activity	UK Biobank questionnaire at baseline; IPAQ activity group divides group into Low, Moderate and High values.	22032
The use of glucocorticoid	UK Biobank questionnaire at baseline; the status of this variable was categorized as using one or more than one glucocorticoid, or not.	1140874816, 1141167174, 1141174548, 1140874790, 1140882622, 1140882764, 1140882766, 1140882774, 1140882780, 1141179982, 1140868364, 1140874930, 1140874976, 1140883026, 1141157402, 1140874896, 1140876456, 1140878562, 1140879922, 1140879934, 1140882822, 1140882824, 1140882830, 1140882836, 1140882840, 1140882842, 1140882844, 1140882846, 1140882848, 1140882850, 1140882864, 1140882888, 1140882894, 1140882896, 1140882902, 1140882906, 1140882908, 1140882914, 1140882918, 1140884672, 1140884704, 1140888134, 1140910424, 1140910634, 1141157294, 1141173346, 1141181062, 1141181610, 1141189464, 1141194840
